# HPLC-MS Detection of Nonylphenol Ethoxylates and Lauryl Ethoxylates in Foodstuffs and the Inner Coatings of High-Barrier Pouches

**DOI:** 10.3390/foods14162842

**Published:** 2025-08-16

**Authors:** Monika Beszterda-Buszczak, Magdalena Frańska, Rafał Frański

**Affiliations:** 1 Department of Food Biochemistry and Analysis, Poznań University of Life Sciences, Mazowiecka 48, 60-623 Poznań, Poland; monika.beszterda@gmail.com; 2 Institute of Chemistry and Technical Electrochemistry, Poznań University of Technology, Berdychowo 4, 60-965 Poznań, Poland; magdalena.franska@put.poznan.pl; 3 Faculty of Chemistry, Adam Mickiewicz University, Uniwersytetu Poznańskiego 8, 61-614 Poznań, Poland

**Keywords:** high-barrier pouches, non-ionic surfactants, nonylphenol ethoxylates, ethoxylated lauryl alcohol, liquid chromatography, electrospray ionization mass spectrometry

## Abstract

The widespread use of non-ionic surfactants, e.g., nonylphenol ethoxylates or dodecyl ethoxylates, may result in their occurrence in foodstuffs. In this paper, extracts from the coatings and from the contents of high-barrier food pouches were analyzed by high-pressure liquid chromatography–mass spectrometry. These flexible pouches are an alternative package format of growing interest which can replace traditional cans. In almost all samples, nonylphenol ethoxylates and dodecyl ethoxylates were detected. The identified nonylphenol ethoxylates usually contained 4–10 oxyethylene units, while the identified dodecyl ethoxylates contained 3–13 oxyethylene units. However, in a few samples, longer fractions of dodecyl ethoxylates were detected, namely those containing >15 oxyethylene units. A comparison of the non-ionic surfactant concentrations in the coating extracts with their concentrations in the content extracts indicated that the coating materials were not the main sources of the contents’ contamination. Other contaminants, namely BADGE conjugates and cyclic cooligoesters, which are common contaminants of canned foodstuffs, were found to rarely occur in high-barrier food pouches. Unexpectedly, in one sample polypropylene glycol was detected at a low concentration; this compound has not been previously identified as a potential food contaminant.

## 1. Introduction

Alkylphenol and alcohol ethoxylates are common non-ionic surfactants which have been used for various industrial and domestic applications, e.g., as detergents, emollients, wetting agents, emulsifiers, and degreasers. The widespread use of these compounds is responsible for their occurrence in the environment, which has stimulated the development of analytical methods for effectively detecting these compounds in the environment [1,2,3], monitoring the distribution of these compounds and their degradation products in various environmental systems [4,5,6,7], and evaluating the environmental risk related to their occurrence [8,9,10,11,12].

It is also already known that the widespread use of non-ionic surfactants may lead to their occurrence in foodstuff. Nonylphenol ethoxylates (NPEO_n_) are among the most common non-ionic surfactants, and nonylphenol (NP) is the product of their degradation [13]. Consequently, there are a number of papers concerning the analysis of nonylphenols in food samples [14,15,16,17,18,19,20,21,22,23,24,25]. Papers analyzing NPEO_n_ concentrations in food samples are less numerous than those concerning NP analysis [26]. Jiang et al. analyzed NPEO_n_ in three leafy vegetables (cabbage, lettuce, and spinach) [27], and She at al. analyzed short NPEO_n_ (NPEO_2_ and NPEO_1_) in various kinds of cultivated plants (cabbage, leek, cucumber, maize, and soy bean) [28]. Cai et al. analyzed NP and NPEO_1_ in ten kinds of vegetables [29]. Chen and Mullin analyzed the presence of NPEO_n_ in honey [30]. These four papers have provided evidence of NPEO_n_ entering food samples from the environment [27,28,29,30]. Nonylphenol ethoxylates may also be introduced to food from food packaging materials. Barahona et al. found NP and short NPEO_n_ (NPEO_2_ and NPEO_1_) in powdered infant milk formula [31]. Viñas et al. detected short NPEO_n_ (NPEO_2_ and NPEO_1_) in commercial fruit juices (orange, pineapple, apple, peach, and grapefruit) [32], while Rashed and Guenther detected nonylphenol carboxylates (NPEO_n_ degradation products) in various foodstuffs [33].

Recently, there has been growing interest in flexible pouches as an alternative package format which can replace traditional cans. Currently, it is difficult to find alternative solutions to high-barrier pouches. They offer a range of important advantages, such as ease of use, relatively low cost, and fewer technical requirements for production. They also provide a broad range of protections, including mechanical protection against impacts and friction; physical protection against sunlight, UV rays, and odors; and protection against potential chemical or microbial contamination. Consequently, multilayer plastic packaging is widely used for various types of food packaging, particularly for baby food pouches and ready-to-serve/microwavable meals. There are numerous studies concerning the properties of packaging materials (e.g., the effects of pressure-assisted thermal sterilization processing on the barrier properties of food pouches) and the chemical, microbiological, and sensory stability of food stored in this type of packaging [34,35,36,37,38,39,40,41,42,43,44]. Therefore, this topic seems to be already quite well researched. On the other hand, packaging of this type may also be a source of potential food contaminants (migrants) and, in contrast to the numerous studies on migrants originating from traditional cans (e.g., devoted to migrant analysis, factors affecting the migration process, and the risk for humans) [45,46,47,48], to the best of our knowledge, flexible pouches have not yet been studied as a source of potential migrants. Therefore, we decided to carry out an HPLC-MS analysis of extracts from the coatings and from the contents of high-barrier pouch foodstuff as a first screening for potential migrants in this type of food packaging.

In this paper, we present the results of our HPLC-MS analysis of non-ionic surfactant concentrations in extracts from the coatings (samples 1A–12A) and from the contents (samples 1B–12B) of high-barrier pouches (Table 1). In almost all samples, nonylphenol ethoxylates (C_9_H_19_-C_6_H_4_-(OCH_2_CH_2_)_n_-OH, NPEO_n_) and dodecyl ethoxylates (ethoxylated lauryl alcohol, C_12_H_25_-(OCH_2_CH_2_)_n_-OH, DDEO_n_) were detected. The ratio of these compounds in the content and coating samples strongly depended on the type of analyzed foodstuff. Potential migrants typical of traditional cans, i.e., BADGE conjugates and cooligoesters, were found in only two samples.

## 2. Materials and Methods

### 2.1. Analyzed Samples

A total of 12 multilayer high-barrier pouches were collected from supermarkets in western Poland in February 2025. Extracts from the coatings (samples 1A–12A) and from the contents (samples 1B–12B) were analyzed (Table 1).

### 2.2. Sample Preparation

A total of 12 multilayer high-barrier pouches were collected from supermarkets in western Poland in February 2025 (see Table 1 for detailed description). All food products were collected in triplicate and initially stored in their original containers, ensuring they did not exceed their expiry date. Empty containers with a net weight ranging from 80 to 170 g were filled with 20 mL of acetonitrile (Super Purity Solvent, Romil Ltd., Cambridge, UK), and those with a net weight ranging from 250 to 400 g were filled with 50 mL. Mechanical extraction was performed by stirring the empty pouches containing the appropriate volume of acetonitrile using a 6-place stirring block (HS 260 Control, IKA, Staufen im Breisgau, Germany). The sealed packages were attached to a shaker and their contents were gently mixed in a reciprocating motion for 60 min. The extraction procedure was performed twice. The extracts were concentrated by evaporation in a vacuum to a minimum, and the residues were re-dissolved to a final volume of 2 mL with acetonitrile. Prior to HPLC-MS analysis, the samples were filtered again through 0.45 µm syringe filters.

The food samples from the multilayer containers (Table 1) after homogenization were transferred into 50 mL polypropylene centrifuge tubes. In brief, 10 g of the sample was weighed and extracted with 15 mL acetonitrile (Super Purity Solvent, Romil Ltd., Cambridge, UK). Next, 4 g of NaCl and 2 g of MgSO_4_ (both from Avantor Performance Materials, Gliwice, Poland) were added to the solution, which was then vortexed and centrifuged. The top acetonitrile layer (5.0 mL) was carefully transferred to a centrifuge tube and the mixture was centrifuged again. Finally, 2.0 mL of the top layer was filtered through a Millipore PTFE filter (0.22 µm) prior to HPLC-MS analysis.

### 2.3. High-Pressure Liquid Chromatography–Mass Spectrometry Analyses

HPLC-MS analyses were performed using a Waters Arc HPLC pump and a Waters SQD mass spectrometer (single quadrupole-type instrument equipped with an electrospray ionization (ESI) source, Z-spray, Milford, MA, USA). The software used was MassLynx V4.2 SCN1046 (Milford, MA, USA). The sample solutions were injected into an XBridge^®^ C18 column (3.5 µm, 100 mm × 3 mm i.d.; Waters, Warsaw, Poland) using an autosampler. The injection volume was 10 µL. The solutions were analyzed using a linear gradient of CH_3_CN/H_2_O with a flow rate of 0.6 mL/min. The gradient started from 0% CH_3_CN to 97% H_2_O with 3% of a 10% solution of formic acid in water (in other words, the 10% solution of HCOOH was continuously dosed with 3% of the total flow rate), reaching 97% CH_3_CN after 10 min, and the latter concentration was kept for 5 min. The HPLC-MS analysis was performed in the positive ion mode in the *m/z* range 100–1200. Each sample was analyzed three times. The nebulizing and desolvation gas was nitrogen at a flow rate of 100 and 300 L/h, respectively. The source temperature was 120 °C, and the desolvation temperature was 300 °C. The electrospray source potentials were as follows: capillary, 3 kV; lens, 0.5 V; extractor, 4 V; and cone voltage (CV), 50 and 100 V. At the lower value, [M+H]^+^, [M+NH_4_]^+^, and [M+Na]^+^ ions were detected, whereas at the higher value, only [M+Na]^+^ ions were observed.

In order to semi-quantitively evaluate the amount of ethoxylates in the analyzed extracts, Triton X-100 (Sigma-Aldrich, Poznań, Poland; CAS-No 9036-19-5, C_8_H_17_-C_6_H_4_-(OCH_2_CH_2_)_n_-OH), an octylphenol ethoxylate (OPEO_n_), was used as an internal standard. This is a polydisperse mixture of C_8_H_17_-C_6_H_4_-(OCH_2_CH_2_)_n_-OH compounds, and the concentrations of individual compounds are not known. The limit of detection (LOD) of individual compounds was ~0.05 µg/mL, and the limit of quantitation was ~0.15 µg/mL. The linearity range was ~0.1–100 µg/mL, with R^2^ ≈ 0.96 (Triton X-100 concentrations in the analyzed solution). These values depended on the number of oxyethylene units—e.g., for C_8_H_17_-C_6_H_4_-(OCH_2_CH_2_)_5_-OH, the LOD was 0.07 µg/mL, and for C_8_H_17_-C_6_H_4_-(OCH_2_CH_2_)_10_-OH, the LOD was 0.03 µg/mL (which, among others, may be related to the concentrations of individual compounds in the sample of Triton X-100). The analyses were performed in triplicate, and the relative standard deviation did not exceed 7%.

In order to semi-quantitively evaluate the amount of the polypropylene glycol detected in one of the analyzed extracts, polypropylene glycol (Sigma-Aldrich, Poznań, Poland; CAS-No 25322-69-4) was used as a standard.

## 3. Results and Discussion

### 3.1. Qualitative Identification of Analyzed Ethoxylates

**Figure 1:**
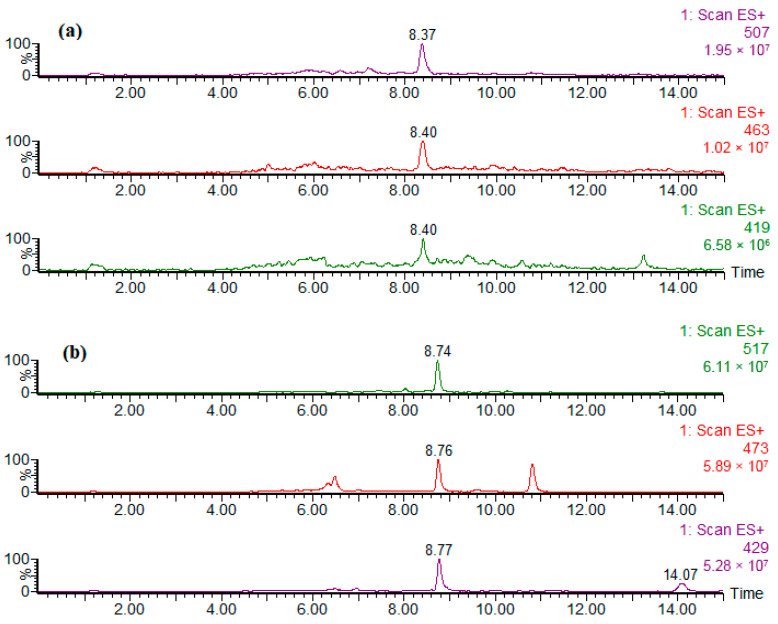
Exemplary single ion chromatograms of [M+Na]^+^ ions obtained upon HPLC-MS analysis of content extracts of sample 7B. [NPEO_4_+Na]^+^ *m*/*z* 419, [NPEO_5_+Na]^+^ *m*/*z* 463, [NPEO_6_+Na]^+^ *m*/*z* 507 (**a**); [DDEO_5_+Na]^+^ *m*/*z* 429, [DDEO_6_+Na]^+^ *m*/*z* 473, [DDEO_7_+Na]^+^ *m*/*z* 517 (**b**).

Figure 1 shows exemplary single ion chromatograms of [M+Na]^+^ ions obtained for the content extract of sample 7B. NPEO_n_ and DDEO_n_ were detected as a series of ions (peaks) differentiated by forty-four Daltons (mass of CH_2_CH_2_O unit) [49,50,51].

**Figure 2:**
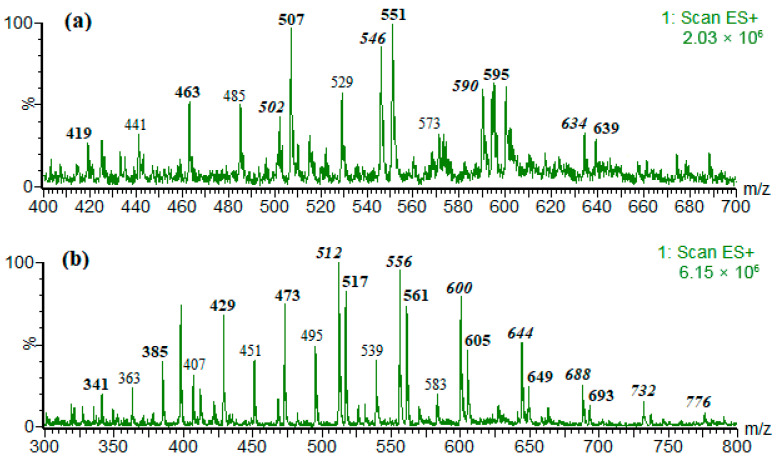
Exemplary ESI mass spectra obtained upon HPLC-MS analysis of content extract of sample 7B (CV = 50 V). (**a**) [NPEO_n_+Na]^+^-*m*/*z* 419 + (44)_n_, [NPEO_n_+NH_4_]^+^-*m*/*z* 458 + (44)_n_, [NPEO_n_+H]^+^-*m*/z 441 + (44)_n_; (**b**) [DDEO_n_+Na]^+^-*m*/*z* 341 + (44)_n_, [DDEO_n_+NH_4_]^+^-*m*/*z* 458 + (44)_n_, [DDEO_n_+H]^+^-*m*/*z* 341 + (44)_n_.

Under the employed chromatographic conditions, the difference of one CH_2_CH_2_O unit caused almost no difference in retention times, while the difference of a few CH_2_CH_2_O units caused slight differences in retention times. Therefore, upon HPLC-MS analysis, it was possible to obtain the ESI mass spectrum containing characteristic peaks separated by 44 Daltons, as shown in Figure 2. It can be assumed that [M+H]^+^ ions are formed from [M+NH_4_]^+^ ions by the loss of a NH_3_ molecule as a result of ‘in-source’ fragmentation. It is clear that the larger the ions are, the less efficient the process is; thus, the larger the ions, the higher the relative abundances of [M+NH_4_]^+^ ions (Figure 2).

In most of the analyzed samples, the identified NPEO_n_ compounds were those with n = 4–10, and the DDEO_n_ compounds were those with n = 3–13 (Figure 2). In samples 7A/7B, 8A, and 9A, longer fractions of DDEO_n_ were detected (n > 10), especially in sample 9A, in which the detected DDEO_n_ compounds were those with n = 16–21. Exemplary mass spectra are shown in the Supplementary Materials (Figure S1).

### 3.2. Semi-Quantitative Analysis of NPEO_n_ and DDEO_n_

Due to the polydispersity of the analyzed compounds, their exact quantitative determination is very difficult (if possible at all) since it would require employment of pure homologous NPEO_n_ and DDEO_n_ molecules. Therefore, it was not possible to carry out methodological assessments such as those of sample recovery, linear range, detection limit, and quantification limit. On the other hand, we decided to use Triton X-100 (octylphenol ethoxylate, OPEO_n_) as an internal standard to estimate the concentrations of the detected NPEO_n_ and DDEO_n_ in the analyzed extracts. Their concentrations were estimated by comparing the extracted ion chromatogram peak areas of NPEO_n_ and DDEO_n_ with those of OPEO_n_ containing an identical number of CH_2_CH_2_O units, n (sums of the peak areas of ions [M+H]^+^, [M+NH_4_]^+^, [M+Na]^+^ were taken for calculations). It can be assumed that the ethoxylate ESI response mainly depends on the ability of ethoxylates to form [M+H]^+^, [M+NH_4_]^+^, and [M+Na]^+^ ions. Therefore, for a given n, NPEO_n_, DDEO_n_, and NPEO_n_ have very similar ESI responses. The hydrophobic parts also affect the ESI response; however, for the analyzed compounds, these parts are of similar sizes (C_15_H_23_-, C_12_H_25_-, and C_14_H_21_-, respectively), so they do not yield significant differences in ESI responses.

**Figure 3:**
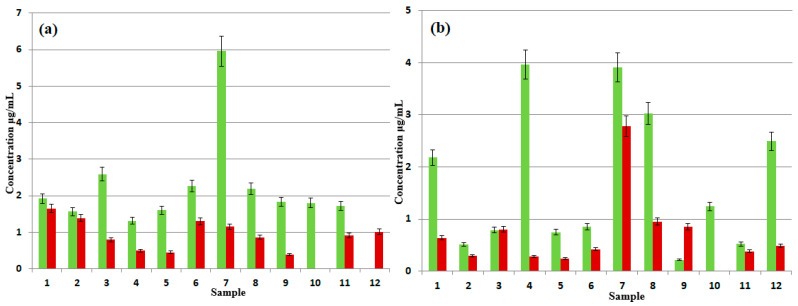
The estimated concentrations (μg/mL) of NPEO_n_ (**a**) and DDEO_n_ (**b**) in the coating extracts (1A–12A, green) and content extracts (1B–12B, red).

Figure 3 shows the estimated concentrations (μg/mL) of NPEO_n_ (Figure 3a) and DDEO_n_ (Figure 3b) in the coating extracts (green) and content extracts (red). Although the food contact material seems to be the main source of non-ionic surfactants in pouch contents, the obtained results may cast doubt on this claim. The coating extract 7A contained the highest amount of NPEO_n_ (~6 μg/mL), whereas in the content extract 7B, the amount of NPEO_n_ was much lower (~1 μg/mL, Figure 3a). An analogous situation was noted for DDEO_n_ in the coating/content extracts of sample 4 (Figure 3b). On the other hand, both the coating and content extracts of sample 7 contained a high amount of DDEO_n_ (~4 and ~3 μg/mL, respectively, Figure 3b). It cannot be expected that the migration degrees of NPEO_n_ and DDEO_n_ are much different; therefore, besides the food contact material, there must be another source of DDEO_n_ in the content extract 7B (Figure 3). In the content extract 12B, the amount of NPEO_n_ was low (~1 μg/mL), but in the coating extract 12A, no NPEO_n_ was detected (its amount was below the detection limit). An analogous situation was noted for DDEO_n_ in the coating and content extracts of sample 9, where the DDEO_n_ amount was much lower in the coating extract 9A than in the content extract 9B (~0.2 and ~0.8 μg/mL, respectively, Figure 3b). It cannot be expected that the NPEO_n_ migration degree in sample 12 and the DDEO_n_ migration degree in sample 9 would be much higher than the migration degree in other samples. Therefore, it is reasonable to assume that besides the food contact material, there must be other sources of NPEO_n_ and DDEO_n_ in the contents of samples 12 and 9, respectively. The coating extract 12A contained a much higher amount of DDEO_n_ than the content extract 12B (~2.5 and ~0.5 μg/mL, respectively, Figure 3b), whereas, as described above, for NPEO_n_ in sample 12, the opposite situation was noted (Figure 3a). Since it is reasonable that the migration degrees of NPEO_n_ and DDEO_n_ are similar, it is another indication that there must be other sources of non-ionic surfactants in the analyzed high-barrier pouch contents. It is also worth noting that both NPEO_n_ and DDEO_n_ were detected in the coating extract 10A, but not in the content extract 10B (Figure 3).

**Figure 4:**
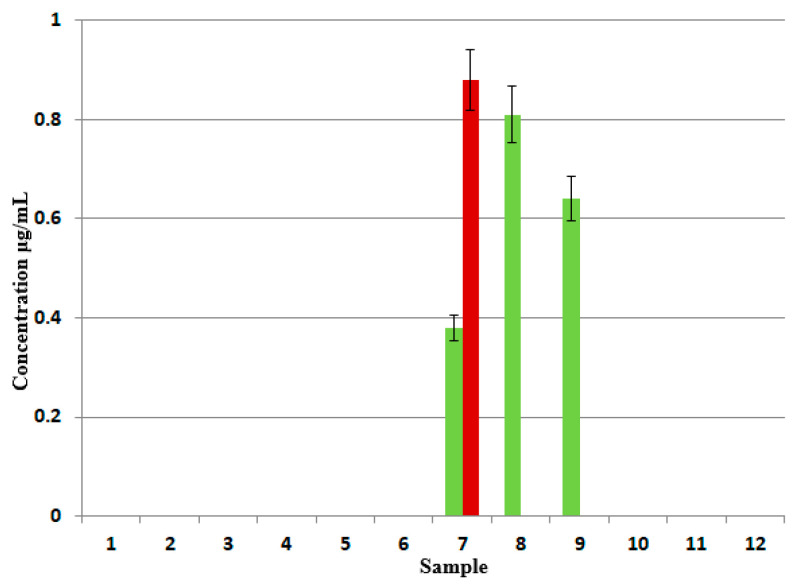
The estimated concentrations (μg/mL) of longer DDEO_n_ fractions in samples 7A/7B, 8A, and 9A (green—coating extracts; red—content extracts).

In four analyzed samples, namely 7A/7B, 8A, and 9A, longer fractions of DDEO_n_ were detected (n > 10). Their estimated amounts were low (<1 μg/mL), and the highest amount was detected in the content extract 7B, which was higher than that for the coating extract 7A, as shown in Figure 4. For samples 8A and 9A, the longer fractions were detected only in the coating extracts. The results obtained for longer DDEO_n_ fractions confirm the earlier mentioned suggestions that the food contact material is not always the source, or not the only source, of non-ionic surfactants in high-barrier pouches’ contents.

Taking into account the packaging types, it is difficult to correlate the amounts of NPEO_n_ and DDEO_n_ determined in the coating extracts with the packaging types. For example, the highest amount of NPEO_n_ was found in sample 7A, one of the coating extracts of a spouted high-barrier pouch, whereas in the other extracts of spouted high-barrier pouches (1A, 2A, 3A, and 5A; Table 1), the amounts of NPEO_n_ were much lower (Figure 3a).

Relatively high amounts of DDEO_n_, namely >2 μg/mL, were found in samples 1A and 7A (extracts of spouted high-barrier pouches), 8A (extract of a multilayer bag with a degassing valve), and 4A and 12A (extracts of retort pouches). Therefore, these three types of packaging can be regarded as a source of DDEO_n_ in food. On the other hand, the amount of DDEO_n_ was also relatively high only in extract 7B (ketchup) (Figure 3b). Since DDEO_n_ migration into the foodstuff in sample 7 is not expected to be much higher than in samples 1, 4, 8, and 12 (Table 1), the coating material in sample 7 most probably was not the source of DDEO_n_.

### 3.3. Comparison of the Oxyethylene Chains Length of the Ethoxylates Detected in the Analyzed Samples

When HPLC-MS analysis was performed at the higher cone voltage value, only [M+Na]^+^ ions were detected. Therefore, the mass spectra obtained in these conditions, which are not affected by the fragmentation/formation of [M+H/NH_4_]^+^ ions, were used for comparison of the relative abundances of detected ethoxylates, containing oxyethylene chains of various lengths.

**Figure 5:**
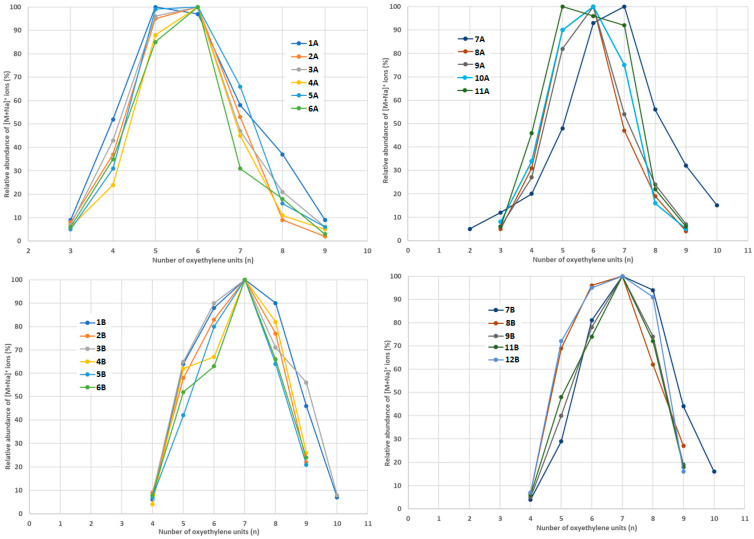
The relative abundances of [M+Na]^+^ ions, where M stands for NPEO_n_ molecules detected in samples 1A–12A (coating extracts) and 1B–12B (content extracts).

**Figure 6:**
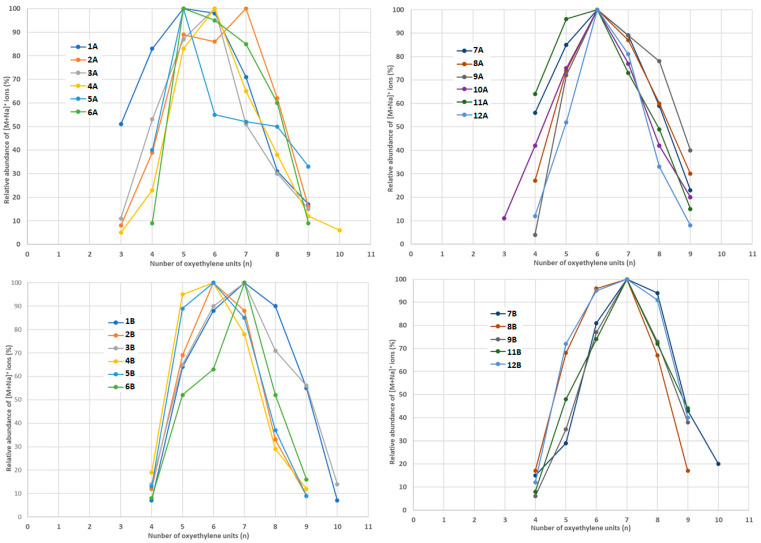
The relative abundances of [M+Na]^+^ ions, where M stands for DDEO_n_ molecules, detected in samples 1A–12A (coating extracts) and 1B–12B (content extracts).

**Figure 7:**
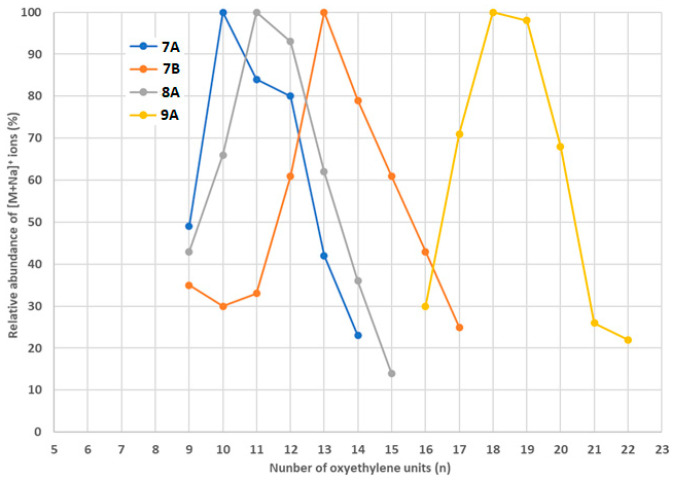
The relative abundances of [M+Na]^+^ ions, where M stands for longer-fraction DDEO_n_ molecules.

Figure 5, Figure 6 and Figure 7 show the relative abundances of [M+Na]^+^ ions, where M stands for an ethoxylate molecule with different oxyethylene chain lengths (containing different numbers of oxyethylene units).

In the content extracts, the most abundant compound was always NPEO_7_, but there were significant differences between the samples with respect to the relative amounts of shorter and longer NPEO_n_ compounds (Figure 5). The abundant longer NPEO_n_ compounds were found in the content samples 1B, 7B, and 12B; however, these samples drastically differed in composition. Therefore, it was also difficult to find a correlation between the analyzed contents (Table 1) and oxyethylene chain length. On the other hand, it is worth noting that the content extracts contained slightly longer NPEO_n_ compounds than the coating extracts (Figure 5).

In the coating extracts, the most abundant compound was usually DDEO_6_, with the exception of samples 2A and 1A/6A, in which the most abundant compounds were DDEO_7_ and DDEO_5_, respectively (Figure 6). In the content extracts, the most abundant compound was usually DDEO_7_, with the exception of samples 4B and 5B, in which the most abundant compound was DDEO_7_ (Figure 6). As with NPEO_n_, it was difficult to find a correlation between the oxyethylene chain length of the detected DDEO_n_ and the type of analyzed samples (Figure 6, Table 1). Furthermore, the content extracts contained slightly longer DDEO_n_ compounds than the coating extracts (Figure 6).

It is clear that there are significant differences in the analyzed samples with respect to the abundances of the detected ethoxylates with a given number of oxyethylene units. In the coating extracts, the most abundant compounds were NPEO_n_ with n = 5 or 6, with the exception of sample 7A, in which the most abundant compound was NPEO_7_ (Figure 5). It is worth noting that sample 7A contained the highest amount of NPEO_n_ (Figure 3). On the other hand, it is difficult to find a correlation between packaging type (Table 1) and oxyethylene chain length.

The longer fractions of DDEO_n_ found in samples 7A/7B, 8A, and 9A also show differences with respect to the abundances of the detected ethoxylates with a given number of oxyethylene units. The longest fraction was found in sample 9A, in which the most abundant compounds were DDEO_18_ and DDEO_19_ (Figure 7). The significant differences between samples 7A and 7B suggest that the longer fractions of DDEOn found in these samples are not of the same origin.

### 3.4. Detection of Other Potential Migrants in the High-Barrier Pouch Coating Material

In three of the analyzed samples of high-barrier pouches, besides the above described non-ionic surfactants, other kinds of potential migrants were detected, as shown in Table 2.

In sample 5A (extract of a spouted high-barrier pouch), cyclic cooligoesters, consisting of one diol monomer, namely neopentyl glycol (NPG), and adipic acid/isophthalic acid (AA/iPA) as diacid comonomers, were detected as a series of ions (peaks) differed by twenty Daltons (Figure S2). Identification of cooligoesters in sample 5A indicates that the food contact material in sample 5 contains polyesters. The detailed mass spectrometric identification of these compounds has been described elsewhere [52,53]. It has already been established that cyclic oligoesters are more toxic than linear ones since the former are usually assessed as Cramer III compounds [54]; therefore, spouted high-barrier pouches may be regarded as a potential source of relatively dangerous compounds. However, from among the five extracts of spouted high-barrier pouches (Table 1), cyclic cooligoesters were detected only in one extract (5A).

In sample 9A (extract of a high-barrier pouch/filter paper), polypropylene glycol (PPG) was detected (concentration ~5 μg/mL) as a series of ions (peaks) differed by fifty-eight Daltons (Figure S3). It is a slightly surprising result since, to the best of our knowledge, this compound has not been identified previously as a potential migrant. Sample 9A was the only extract of a high-barrier pouch/filter paper; therefore, further studies may be desirable to determine if PPG is a more common compound occurring in this type of packaging (although PPG toxicity is very low [55]).

In sample 12A (extract of a retort pouch), conjugates of bisphenol A di(mono)glycidyl ether (BAD(M)GE) with butoxyethanol (BuOEtOH) were identified on the basis of characteristic product ions [56,57]. Identification of these conjugates in sample 12A indicates that the food contact material in sample 12 contained bisphenol A-based epoxy resin. The most abundant conjugates were BADGE + BuOEtOH + H_2_O, BAMGE + BuOEtOH, and BADGE + 2BuOEtOH (Figure S4). This type of contaminant is one of the most common which occurs in traditional can coating material and canned foods (epoxy resin), and there is one prior example of a BADGE conjugates analysis in retort pouches [58].

## 4. Conclusions

The results of the performed HPLC-MS analysis have shown that non-ionic surfactants, namely nonylphenol ethoxylates and ethoxylated lauryl alcohol, are the main contaminants of high-barrier pouch foodstuffs. Their estimated concentrations in the analyzed samples were rather low; thus, the possible risk for consumer health seems to be also low. The comparison of their concentrations in the coating extracts with those in the content extracts indicated that the coating materials are not the main sources of the content contamination. Therefore, it is reasonable to suppose that non-ionic surfactants enter the content at some stage of foodstuff production. On the other hand, the release of these compounds from the coating materials into the content cannot be definitely excluded (the compounds are well soluble probably in each solvent). It is expected that the compounds containing fewer oxyethylene units, being more lipophilic, can better migrate into fat-containing products, whereas the compounds containing more oxyethylene units, being more hydrophilic, can better migrate into fruit products. Other contaminants, e.g., BADGE conjugates or cyclic cooligoesters (typical of canned foodstuffs), were found to rarely occur in high-barrier pouch foodstuffs, and they were detected only in coating extracts. In summary, high-barrier pouch foodstuffs seem to be relatively safe for consumer health. This may be the reason why this type of foodstuff has not yet been widely studied with respect to potential migrants.

## Figures and Tables

**Table 1:** List of analyzed high-barrier pouch samples: A—coating extracts; B—content extracts.

Sample	Description	Packaging Type	Protein (%)	Fat (%)	Ingredients
1A, 1B	Fruit puree	Spouted high-barrier pouch	<0.5	<0.5	Apple, banana, lemon juice, ascorbic acid
2A, 2B	Fruit puree	Spouted high-barrier pouch	0.5	0.4	Apple, peach, mango, ascorbic acid
3A, 3B	Fruit puree	Spouted high-barrier pouch	1.6	1.1	Banana, mango, yoghurt (milk, water, skimmed milk powder, pectin, milk yoghurt cultures), water, rice flour, oat flour, lemon juice, ascorbic acid
4A, 4B	Tomato puree	Retort pouch	1.3	0	Tomato purée, salt
5A, 5B	Mustard	Spouted high-barrier pouch	6.9	7.8	Water, mustard seed, sugar, white mustard seed, mustard powder, table salt, acetic acid, refined deodorized sunflower oil, curcumin, xanthan gum, sodium benzoate, mustard flavor, sodium metabisulfite, paprika extract, ground cloves, turmeric extract
6A, 6B	Hungarian goulash soup	Retort pouch	3.6	1.6	Water, paprika, potatoes, onion, pork, modified starch, tomato concentrate, wheat flour, salt, pepper, thyme, parsley, yeast extract, sodium ascorbate
7A, 7B	Ketchup	Spouted high-barrier pouch	1.2	0	Water, tomato paste, sugar, modified corn starch, salt, acetic acid, sweet paprika, onion, garlic, chili pepper, potassium sorbate, cinnamon, black pepper, cloves
8A, 8B	Roasted coffee beans	Multilayer bag with degassing valve	0	0	Roasted Arabica coffee beans
9A, 9B	Roasted coffee beans—Bagdrip	High-barrier pouch/filter paper	0	0	Roasted Arabica coffee beans
10A, 10B	Freeze-dried fruit smoothie	High-barrier pouch	5.9	2.0	Apple, kiwi, pineapple, spinach, nettle, ginger
11A, 11B	Freeze-dried granola with strawberries	High-barrier zipper pouch	11	5.1	Gluten-free oatmeal, agave syrup, strawberry, cocoa
12A, 12B	Freeze-dried wholemeal bread	Retort pouch	5.2	1.2	Wholemeal rye flour, water, salt, yeast

**Table 2:** Other kinds of potential migrants detected.

Sample	Potential Migrants
5A	Cyclic cooligoesters, (NPG-AA)_n_-(NPG-iPA)_m_, n, m = 0–5
9A	Polypropylene glycol, HO-(CH_2_-CH(CH_3_)-O)_n_-H, n = 4–15
12A	BAD(M)GE conjugates

## Data Availability

The original contributions presented in this study are included in the article/Supplementary Materials. Further inquiries can be directed to the corresponding author.

## Supplementary Materials

The following supporting information can be downloaded at: https://www.mdpi.com/article/10.3390/foods14162842/s1, Figure S1: Exemplary ESI mass spectra of longer DDEO_n_ fractions; Figure S2: Single ion chromatograms of [M+NH_4_]^+^ ions of cyclic cooligoesters (NPG-AA)_n_-(NPG-iPA)_m_ identified in sample 5A; Figure S3: Exemplary single ion chromatograms of [M+NH_4_]^+^ ions of polypropylene glycol identified in sample 9A. Figure S4: Single ion chromatograms of characteristic product ions and obtained ESI mass spectra (sample 12A) of conjugates of bisphenol A di(mono)glycidyl ether (BAD(M)GE) with butoxyethanol (BuOEtOH); BADGE+BuOEtOH+H_2_O [M+H]^+^ *m*/*z* 477, BAMGE+BuOEtOH [M+H]^+^ *m*/*z* 402, BADGE+2BuOEtOH [M+H]^+^ *m*/*z* 577.
