# Supplementary material for: HPLC-MS Detection of Nonylphenol Ethoxylates and Lauryl Ethoxylates in Foodstuffs and the Inner Coatings of High-Barrier Pouches
Source: Foods. 2025 Aug 16;14(16):2842. doi: 10.3390/foods14162842 (PMC12385684; doi:10.3390/foods14162842)
Supplement: Supplementary file 1 [file foods-14-02842-s001.zip › foods-3744080-supplementary.pdf]

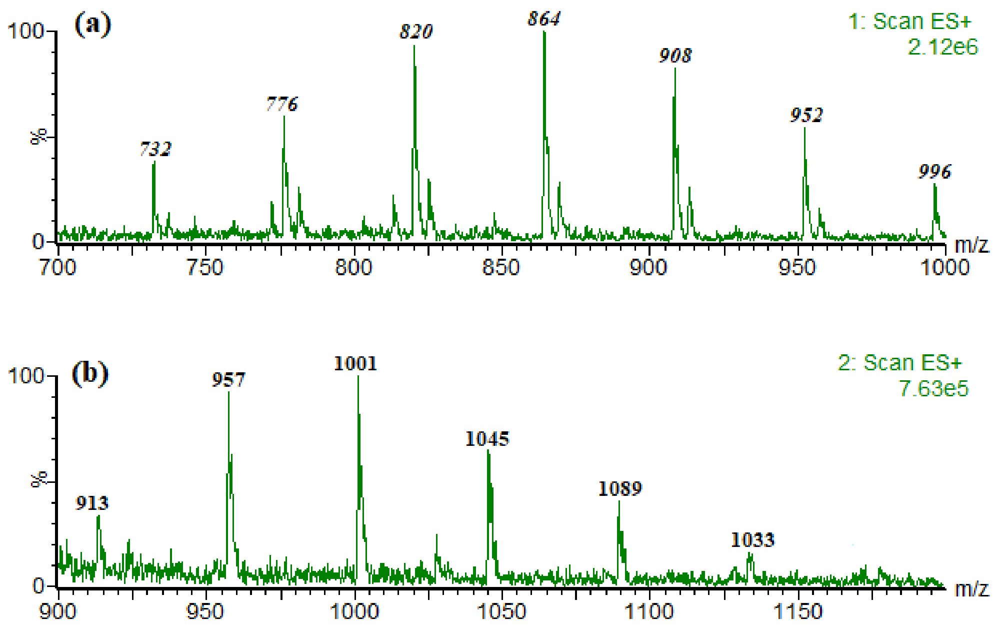


**Figure S1.** Exemplary ESI mass spectra of $DDEO_n$ longer fractions. **(a)** Sample 7B, CV = 50 V, ions $[DDEO_n+NH_4]^+$ with n = 12-18; **(b)** Sample 7B, CV = 100 V, ions $[DDEO_n+Na]^+$ with n = 16-21.

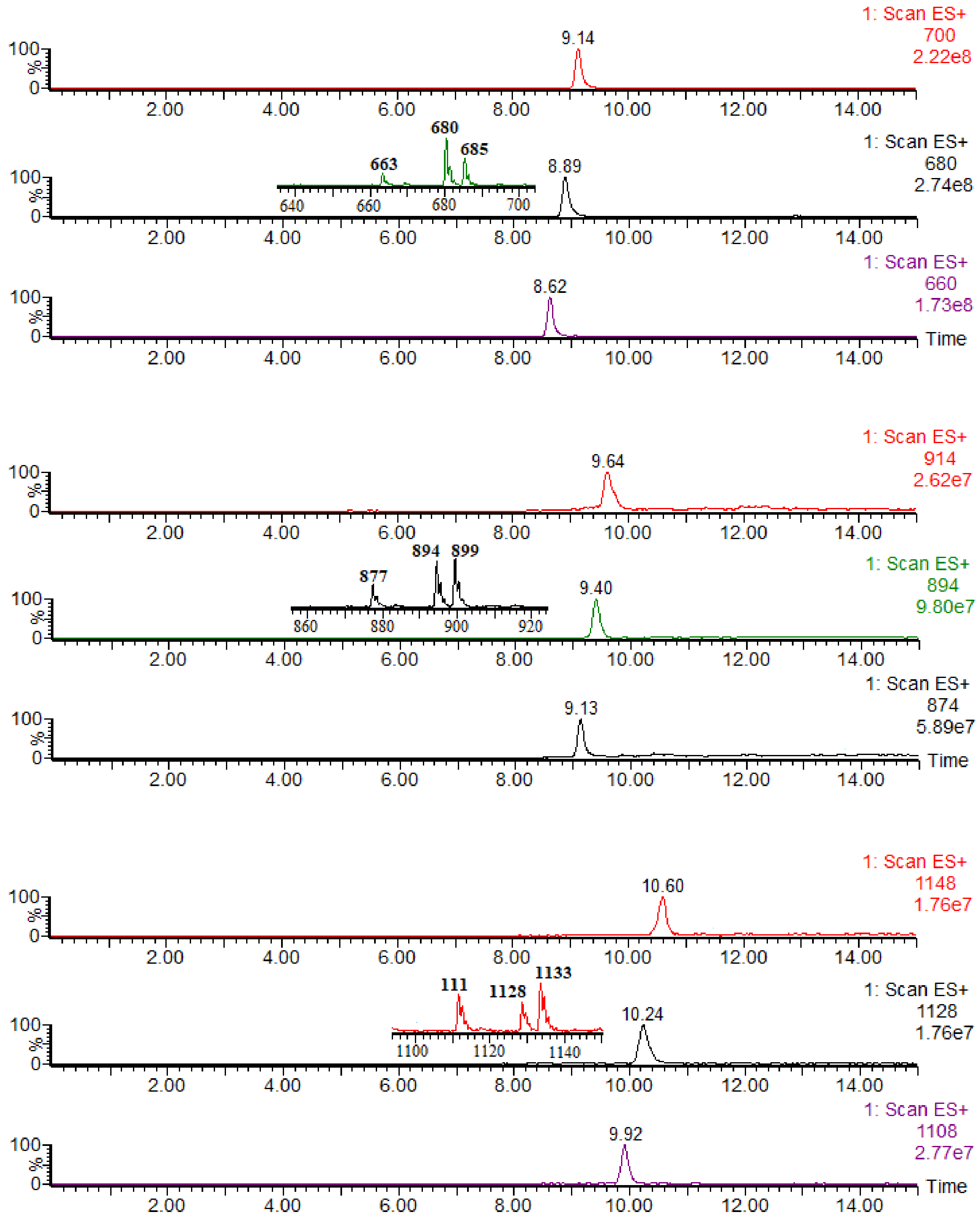


**Figure S2.** Single ion chromatograms of $[M+NH_4]^+$ ions of cyclic cooligoesters, $(NPG\text{-}AA)_n$-$(NPG\text{-}iPA)_m$ identified in sample 5A.

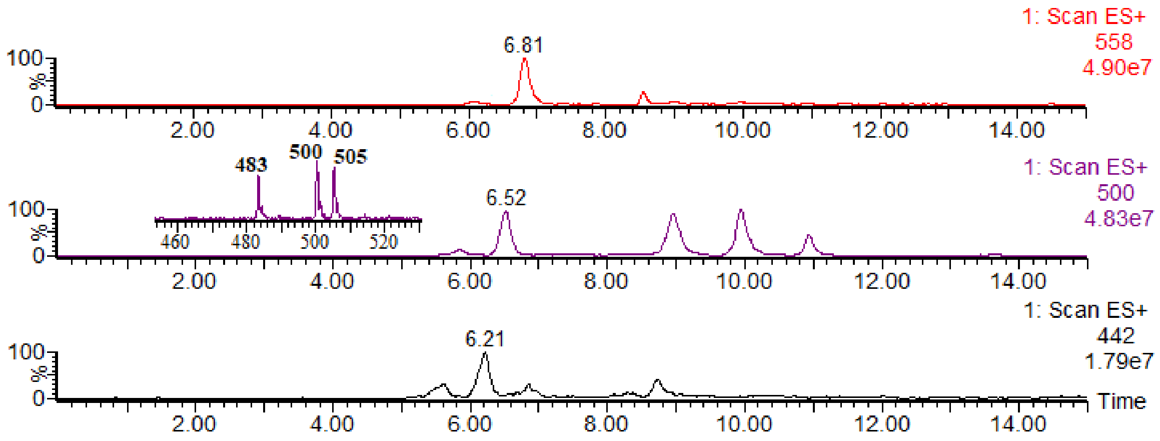


**Figure S3.** Exemplary single ion chromatograms of $[M+NH_4]^+$ ions of polypropylene glycol identified in sample 9A.

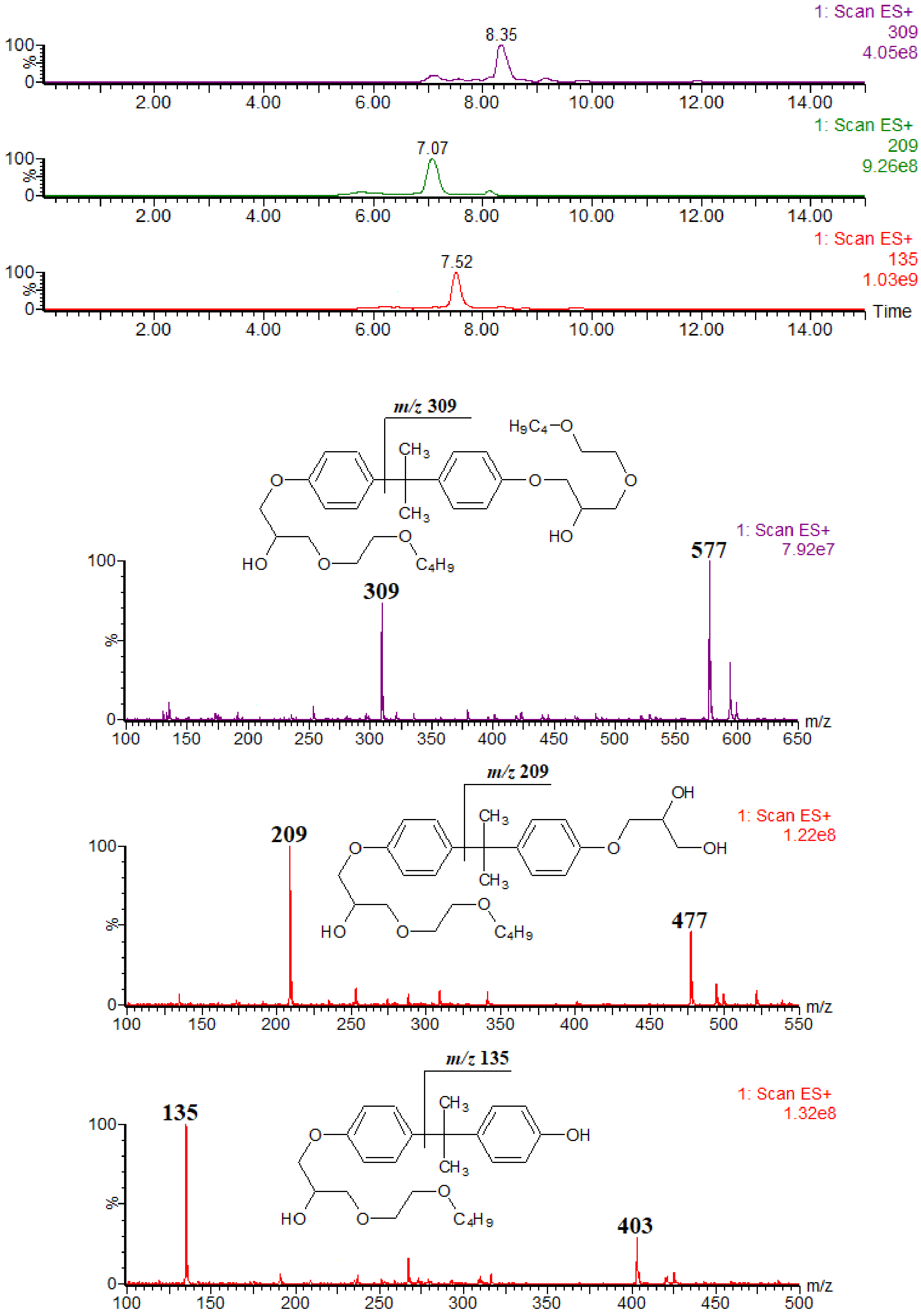


**Figure S4.** Single ion chromatograms of characteristic product ions and obtained ESI mass spectra (sample 12A) of conjugates of bisphenol A di(mono)glycidyl ether (BAD(M)GE) with butoxyethanol (BuOEtOH); BADGE+BuOEtOH+$H_2O$ $[M+H]^+$ *m/z* 477, BAMGE+BuOEtOH $[M+H]^+$ *m/z* 402, BADGE+2BuOEtOH $[M+H]^+$ *m/z* 577.
